# FUSE-PhyloTree: linking functions and sequence conservation modules of a protein family through phylogenomic analysis

**DOI:** 10.1093/bioinformatics/btaf479

**Published:** 2025-08-28

**Authors:** Olivier Dennler, Elisa Chenel, François Coste, Samuel Blanquart, Catherine Belleannée, Nathalie Théret

**Affiliations:** Univ Rennes, Inria, CNRS, IRISA - UMR 6074, Rennes, F-35000, France; Univ Rennes, INSERM, EHESP, Institut de Recherche en Santé Environnement et Travail (IRSET) - UMR_S 1085, 9 avenue du Prof. Léon Bernard, Rennes, F-35000, France; Univ Rennes, Inria, CNRS, IRISA - UMR 6074, Rennes, F-35000, France; Univ Rennes, Inria, CNRS, IRISA - UMR 6074, Rennes, F-35000, France; Univ Rennes, Inria, CNRS, IRISA - UMR 6074, Rennes, F-35000, France; Univ Rennes, Inria, CNRS, IRISA - UMR 6074, Rennes, F-35000, France; Univ Rennes, Inria, CNRS, IRISA - UMR 6074, Rennes, F-35000, France; Univ Rennes, INSERM, EHESP, Institut de Recherche en Santé Environnement et Travail (IRSET) - UMR_S 1085, 9 avenue du Prof. Léon Bernard, Rennes, F-35000, France

## Abstract

**Summary:**

FUSE-PhyloTree is a phylogenomic analysis software for identifying local sequence conservation associated with the different functions of a multi-functional (e.g. paralogous or multi-domain) protein family. FUSE-PhyloTree introduces an original approach that combines advanced sequence analysis with phylogenetic methods. First, local sequence conservation modules within the family are identified using partial local multiple sequence alignment. Next, the evolution of the detected modules and known protein functions is inferred within the family’s phylogenetic tree using three-level phylogenetic reconciliation and ancestral state reconstruction. As a result, FUSE-PhyloTree provides a gene tree annotated with both predicted sequence modules and ancestral gene functions, enabling the association of functions with specific sequence regions based on their co-emergence.

**Availability and implementation:**

FUSE-PhyloTree is provided as Docker and Singularity images including all the required software tools. Images, source code, test data, and documentation are available at https://github.com/OcMalde/fuse-phylotree and https://zenodo.org/records/15855068.

## 1 FUSE-PhyloTree

FUSE-PhyloTree is dedicated to the study of multi-functional protein families, including for instance paralogous or multi-domain proteins. These families, containing proteins with different and possibly multiple functions, are challenging to study experimentally and are often incompletely annotated, with missing functional annotations for some proteins and limited identification of the functionally important sequence regions. While common, these families are difficult to analyze with traditional computational tools due to their complex evolution, which can involve sequence duplication, insertion, and deletion events, along with varying selection pressures on specific sequence regions as functions are gained or lost.

Methods for refining protein families into functional families (FunFams) and predict their functional sites are reviewed by Rauer *et al.* (2021). The first approach relies on detecting known domains (Wang *et al.* 2021), such as those from the integrated database Interpro (Paysan-Lafosse *et al.* 2023). However, the number of known functional domains is limited, and the precision of the predicted annotations can be insufficient for the family functional characterization. Rauer *et al.* (2021) thus highlights two main approaches: (i) constructing sequence similarity network, typically by pairwise comparisons, and (ii) clustering the multiple sequence alignment (MSA) of the family with respect to a phylogenetic tree.

The advantage of the alignment approach (ii) over approach (i) is to consider the entire family sequence information. Yet, proteins within multi-functional families have distinct functions supported by different sequence regions. They are thus difficult to characterize with classical MSA since functionally important regions may be conserved only within specific subsets of the family and may occur at different locations. To deal with such families, the first originality of FUSE-PhyloTree lies in using an original alignment method developed by our team (Kerbellec 2008, Dennler *et al.* 2023): the *partial local multiple alignment* (PLMA). A PLMA identifies all compatible blocks of local sequence conservation shared by at least two sequences, each block consisting in a local alignment of significantly similar segments from some of the sequences to align. These blocks, referred to as sequence conservation modules or simply *modules*, form the basis of our approach.

According to the definition of a PLMA block, identifying a set of modules that encompass all key functional sequence segments requires that each function of interest is represented by at least two sequences in the aligned set. This can be achieved by incorporating orthologs within the family. However, the association of the specific (sets of) modules with the particular functions will still remain unknown. The second originality of FUSE-PhyloTree lies in combining this sequence analysis with a phylogenomic approach (Eisen 1998). While approach (ii) uses phylogenetic inference to cluster sequence alignment, FUSE-PhyloTree proposes to use advanced methods to infer and map the *evolutionary history* of both the *functions* and the *modules* onto the phylogenetic tree of the protein family (which can include paralogs and orthologs). Concerning the functions, FUSE-PhyloTree uses the maximum likelihood method for ancestral state reconstruction proposed by Ishikawa *et al.* (2019) to infer their presence/absence at each ancestral node of the gene tree. With respect to the sequences, estimating the modules’ history is much more complex, as gene regions undergo their own events (gain/loss) independently of their host gene’s history, as well as the genes encounter their own events (paralogous duplication/loss) independently of their host species’ history. To map the modules’ history onto the gene tree, FUSE-PhyloTree uses the reconciliation method by Li and Bansal (2019), which accounts for duplication, transfer, and loss events occurring during both the modules’ evolution and the genes’ evolution.

As a result, FUSE-PhyloTree generates a phylogenetic tree annotated with both the ancestral function and module content of its nodes, enabling the study of their associations. Natural selection preserves the protein sequence regions that are critical for essential functions, and the conservation of functions and modules since a given common ancestor suggests their potential association. By identifying the co-emergence of functions and modules within the annotated gene tree, FUSE-PhyloTree proposes candidate module sets likely to be linked to specific functions. Additionally, FUSE-PhyloTree enables interactive visual exploration of the annotated gene tree with iTOL (Letunic and Bork 2024), enabling in-depth examination of the co-evolution of functions and modules.

In practice, FUSE-PhyloTree identifies sets of non-contiguous, conserved sequence regions (module signatures) that offer finer granularity than traditional domain annotations. These modules are detected independently of known domains. They characterize each sub-family, and can be linked to shared phenotypes or functions (such as PPI annotations, for instance). Applied to the ADAMTS/TSL family (Dennler *et al.* 2023), FUSE-PhyloTree uncovered module signatures that overlap with experimentally identified functional regions, or that clustered in 3D space despite being distant in sequence. In ADAMTS-5, e.g. they captured functionally distinct regions missed by domain-based annotations. Other signatures revealed sub-gene specializations, including convergent interactions across ADAMTS paralogs. We also applied FUSE-PhyloTree to the fibulin family (step-by-step illustration of the analysis is available in the Appendix, available as supplementary data at *Bioinformatics* online). It identified 14 module–PPI signatures, including one specific to the elastic fibulins, consistent with experimental findings and revealing novel conserved regions beyond standard domain boundaries. Overall, these results underline FUSE-PhyloTree’s ability to unravel the complexity of multi-functional protein families by identifying fine-scale, interpretable module signatures and the specific functions they underpin within their respective families.

## 2 Workflow

**Figure 1.**
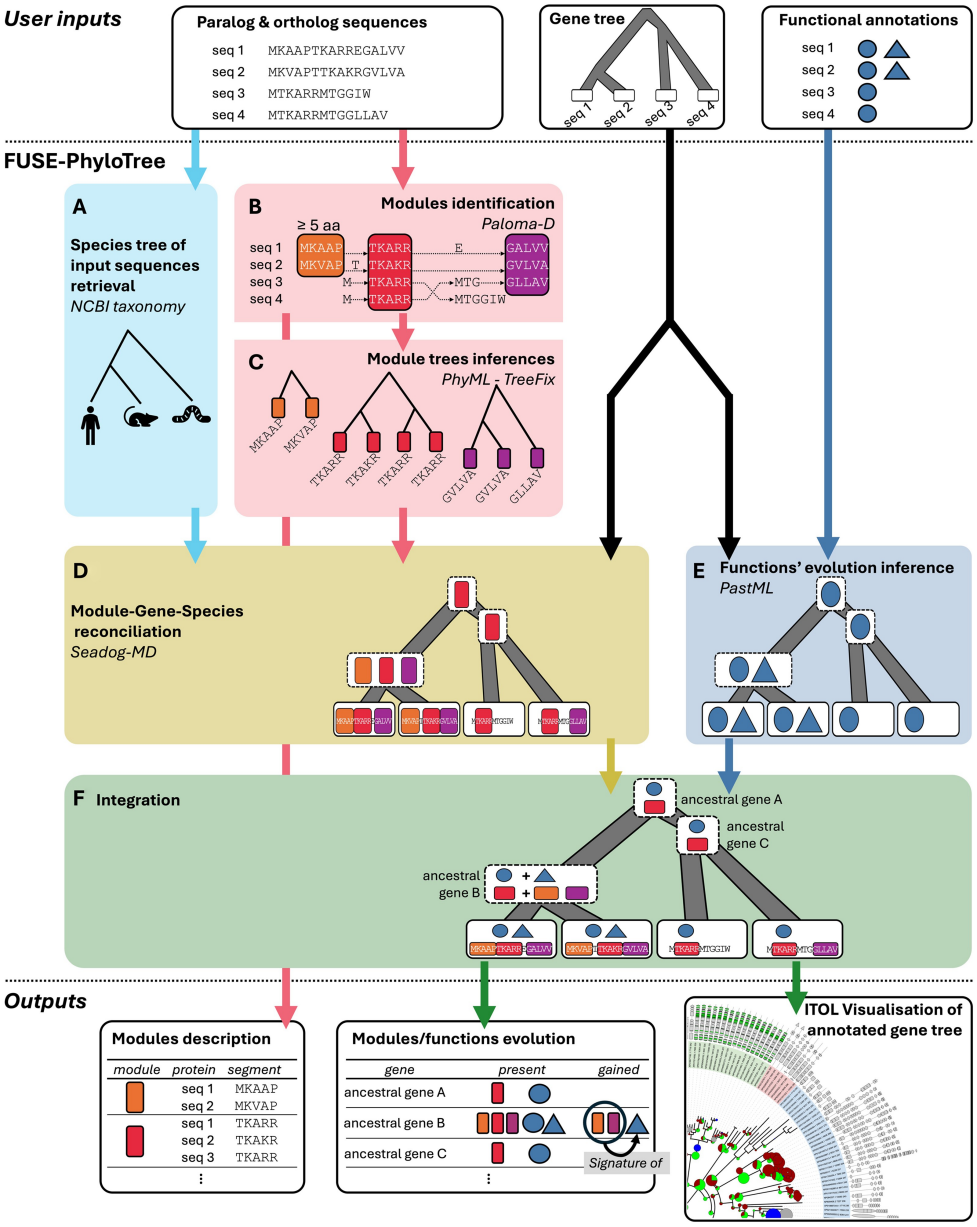
FUSE-PhyloTree workflow. FUSE-PhyloTree takes three input data files: a set of protein sequences (FASTA file), a binary rooted gene tree for these sequences (newick file), and their associated functional annotations (e.g. protein-protein interactions, CSV file). In this schema, there are only two functions symbolized by a triangle and a circle, respectively. (A) The species tree corresponding to the input sequences is retrieved from NCBI taxonomy. (B) Identification of sequence conservation modules using Paloma-D. The boxes indicate the modules, defined by aligned protein segments. (C) For each module, a phylogenetic module tree is computed using PhyML and TreeFix considering the gene tree as a reference. (D) The species, gene and module trees are reconciled using SEADOG-MD, to infer the presence of modules along the gene tree. (E) The evolution of functions along the gene tree is inferred using PastML, considering the gene tree and the protein functional annotations as input and mapping the presence of functions at each tree node. (F) The integration of previous steps yields a gene tree annotated with the presence of both modules and functions, facilitating the exploration of sequence-function associations through their evolutionary history. Output files include interactive iTOL representations (text files—iTOL) and tabular files listing the presence, gain, and loss of modules and functions at each gene node, enabling the identification of putative module signatures of functions through their co-emergence.

Given a multi-functional protein family, FUSE-PhyloTree integrates sequence and phylogenomic analyses to predict functional regions by associating conserved sequence regions with specific functional annotations. We present here the different steps of FUSE-PhyloTree’s pipeline (Fig. 1).

FUSE-PhyloTree takes as input the three following files: (i) a FASTA file containing *protein sequences of the target family (including both paralogs and orthologs)* with the indication of their NCBI taxonomic identifier (*taxid*), (ii) a newick file for *the rooted binary gene tree corresponding to these sequences*, and (iii) a tabular file (CSV format) of *functional annotations* of interest of proteins, for instance their identified protein-protein interactions. By default, FUSE-PhyloTree requires a fully annotated subset of proteins, typically from a well-studied model organism. It then assumes that the absence of an annotation for any of these proteins indicates that the protein does not perform the corresponding function. Protein sequences outside the annotated subset are treated as having undetermined functionality, meaning they may or may not carry out the functions. Users may override this behavior.

FUSE-PhyloTree method involves six key steps: (i) The species tree for the input sequences is retrieved from NCBI taxonomy using their *taxid*. (ii) Sequence conservation modules are identified using Paloma-D (Kerbellec 2008, Dennler *et al.* 2023). (iii) Each module’s independent evolutionary history is inferred as a phylogenetic module tree with PhyML (Guindon *et al.* 2010), corrected using TreeFix (Wu *et al.* 2013) considering the gene tree as template. (iv) Module, gene, and species trees are reconciled using SEADOG-MD (Li and Bansal 2019) to infer modules’ evolution, identifying which modules are present at each ancestral gene node, thereby determining when modules were gained or lost (i.e. present or absent compared to the direct parent gene). Because PhyML and TreeFix are non-deterministic, two runs can yield slightly different evolutionary histories, differing in the inferred ancestral depth of some small modules that are broadly but unevenly distributed. To identify such uncertainties, the sequence of steps (iii) and (iv) is repeated multiple times (10 by default), and the frequency among these runs of the prediction of modules’ presence, gain and loss at ancestral genes is also reported. (v) In parallel, the evolution of functions in the gene tree is inferred using PastML (Ishikawa *et al.* 2019), mapping which functions are present at each ancestral gene node. (vi) Finally, the evolution of sequence conservation modules and functions is jointly mapped onto the gene tree to produce a unified scenario detailing their presence at each ancestral gene node, as well as their gain or loss relative to the parent node.

FUSE-PhyloTree outputs consist in the detailed descriptions of the sequence conservation modules and the annotated gene tree, showing the presence, gain, and loss of these modules and functions. To reveal potential sequence-function relationships, FUSE-PhyloTree proposes associations between sequence regions and functions based on their simultaneous emergence during gene evolution. Specifically, the outputs consist of: (i) a tabular file listing each module, the sequences in which they are found, their positions and corresponding segments, (ii) a tabular file mapping the presence, gain, and loss of modules and functions at different gene nodes on the gene tree, and (iii) all files for iTOL (Letunic and Bork 2024) interactive visualizations of all generated data. Additional data from intermediate steps, such as the PLMA computed by Paloma-D and all probabilities of function presence at various gene nodes computed by PastML, are available in a directory for further analysis if required. A full list of the generated files and their contents is detailed in the GitHub documentation.

By default, most third-party tools are run with their standard settings; however, users can customize these settings when launching the workflow. For more advanced use, users can also provide their own species tree, PLMA, or even all intermediate files before the final integration step. See the GitHub documentation for user instructions and the Appendix, available as supplementary data at *Bioinformatics* online, for an illustration of the application of FUSE-PhyloTree on a practical example.

## 3 Implementation

FUSE-PhyloTree is implemented as a modular pipeline that integrates multiple standalone software tools with custom Python scripts, handling tasks from input/output formatting to newly developed algorithms for integrating their outputs.

FUSE-PhyloTree relies on the following standalone software: MUSCLE (Edgar 2022) for multiple sequence alignment (MSA), trimAl (Capella-Gutiérrez *et al.* 2009) for MSA trimming, PhyML (Guindon *et al.* 2010) for phylogenetic tree inference, TreeFix (Wu *et al.* 2013) for tree correction, Paloma-D (Kerbellec 2008, Dennler *et al.* 2023) for PLMA computation, SEADOG-MD (Li and Bansal 2019) for three-level DGS (Domain-Gene-Species) reconciliation, and PastML (Ishikawa *et al.* 2019) for ancestral scenario reconstruction of functions.

For standard use, FUSE-PhyloTree is provided as Docker and Singularity images, bundling all required software, dependencies, and scripts for easy use across Linux, Windows and MacOS systems and high-performance computing clusters. This setup allows users to run the pipeline with minimal configuration. All custom Python scripts are freely available in the GitHub repository.

## 4 Conclusion

FUSE-PhyloTree offers a pipeline for exploring sequence conservation modules and their association with functional annotations through phylogenomic analysis. This is the first tool to simultaneously exploit a new method of partial local multiple alignment, three-level phylogenetic reconciliation methods, and ancestral reconstruction of protein functions. This comprehensive framework enables in-depth analysis of sequence-function relationships in multi-functional protein families.

## Supplementary Material

btaf479_Supplementary_Data

## Data Availability

The FUSE-PhyloTree Docker and Singularity images, along with all scripts, test data, and documentation, are available at https://github.com/OcMalde/fuse-phylotree and https://zenodo.org/records/15855068.
