# Supplementary material for: FUSE-PhyloTree: linking functions and sequence conservation modules of a protein family through phylogenomic analysis
Source: Bioinformatics. 2025 Aug 28;41(9):btaf479. doi: 10.1093/bioinformatics/btaf479 (PMC12648394; doi:10.1093/bioinformatics/btaf479)
Supplement: btaf479_Supplementary_Data [file btaf479_supplementary_data.zip › Supplementary_Appendix.pdf]

APPENDIX of *FUSE-PhyloTree: Linking functions and sequence conservation modules of a protein family through phylogenomic analysis*

Illustrated use-case of FUSE-PhyloTree on the fibulin protein family

Olivier Dennler, Elisa Chenel, François Coste, Samuel Blanquart, Catherine Belleannée and Nathalie Théret

We present here a practical example of the application of FUSE-PhyloTree to a multi-domain, multifunctional protein family: the *fibulins* (Cooley and Argraves, 2011).

Fibulins is a family of extracellular matrix glycoproteins associated with basement membrane and elastic fibers. Eight fibulin paralogs are identified in humans and are characterized by variable lengths and domain compositions, including a fibulin-type C-terminal domain preceded by tandem calcium-binding epidermal growth factor (EGF)-like domains (Mahajan et al., 2021). Their expression is altered in many diseases such as cancer where they are thought to play both anti- and pro-oncogenic roles depending on their molecular interaction with other proteins (Gallagher et al., 2005). The identification of specific functional sequence regions within fibulins is therefore promising for the development of new targeted therapeutic strategies.

In this appendix, we consider the different protein-protein interactions of the fibulins as functions of interest, and we illustrate step-by-step how to use FUSE-PhyloTree in order to identify and explore the local sequence conservations that may be associated with these interactions.

Download

The first step is to install the tool. FUSE-PhyloTree is available as a Singularity or Docker image, which are available for download on our GitHub (`https://github.com/OcMalde/fuse-phylotree`). We will use here Singularity (version 3.6.3). If not already available in your environment, Singularity needs to be installed (see `https://docs.sylabs.io/guides/3.0/user-guide/installation.html`).

The FUSE-PhyloTree image can be downloaded by executing the following command in a terminal:
`singularity pull https://github.com/OcMalde/fuse-phylotree/releases/download/V1.1.0/fuse-phylotree.sif`

Input

Then we need to prepare the input data. The program takes three different files as input:

- A **set of reference sequences**, in Fasta format, containing the protein sequences of the family (orthologs and paralogs). To enable automatic retrieval of the species tree, FUSE-PhyloTree requires the Fasta identifiers to be in the form >`SeqID_taxid`, where `SeqID` is a unique identifier for the sequence, such as a RefSeq ID, and `taxid` is the taxonomic identifier in the NCBI Taxonomy database. For studies targeting a family of human paralogs, FUSE-PhyloTree provides the `make_orthogroup_fasta.sh` helper script to automatically complete the reference sequences with orthologs (from the pre-calculated orthogroups for nine bilateral species available in the FUSE-PhyloTree image). Starting from a file containing the RefSeq ID of the initial sequences, the script completes the sequences and generates the formatted Fasta file. Using this script with the 8 human fibulins as input, after discarding a suspect short ortholog that does not contain the required fibulin domains, we obtained 59 fibulin sequences in 9 bilaterian species (see Table S1). These sequences, used hereafter as reference sequences, are available in the `fibulin_59.fasta` file (Supplementary Files).
- A **rooted, binary phylogenetic tree**, in Newik format, of the set of reference sequences.
  For our study, we considered members of the LTBP family (latent transforming growth factor (TGF-β)-binding proteins) as close relatives of the fibulins (Robertson et al., 2015) enabling to root the tree. The outgroup sequences consisted of LTBP-1 and LTBP-4 human paralogs and their orthologs from the eight other bilaterian species considered for the reference sequence file. Adding these sequences to the 59 fibulins, a phylogenetic tree was inferred. Its root was manually set and the outgroup was removed using iTOL (Letunic and Bork, 2024). The resulting phylogenetic tree is available in the file `fibulin_tree_root.tree` (Supplementary Files).
- A set of **functional annotations of reference sequences**, in comma-separated value text format (CSV), where each row contains the known functional annotations of interest of a reference sequence in the form:
  `SeqID,Annotation1|Annotation2|Annotation3...|AnnotationN`
  with the first value being the sequence identifier and the second value specifying the annotations of the sequence separated by the | symbol.
  In our use-case, the considered functional annotations are the protein-protein interactions (PPIs) currently known for human fibulins. They were retrieved through the Proteomics Standard Initiative Common Query Interface (PSICQUIC) webservice (Aranda et al., 2011) and filtered to retain only 97 PPIs shared by at least two human fibulin paralogs (Fig. S1). The resulting functional annotation file is `ppi_shared.csv` (Supplementary Files).

Table S1. Number of fibulin paralogs per species

**Species**	Number of sequence
Homo sapiens	8
Mus musculus	8
Bos taurus	8
Gallus gallus	7
Xenopus tropicalis	9
Danio rerio	12
Ciona intestinalis	5
Drosophila melanogaster	0
Caenorhabditis elegans	2
**Total**	**59**

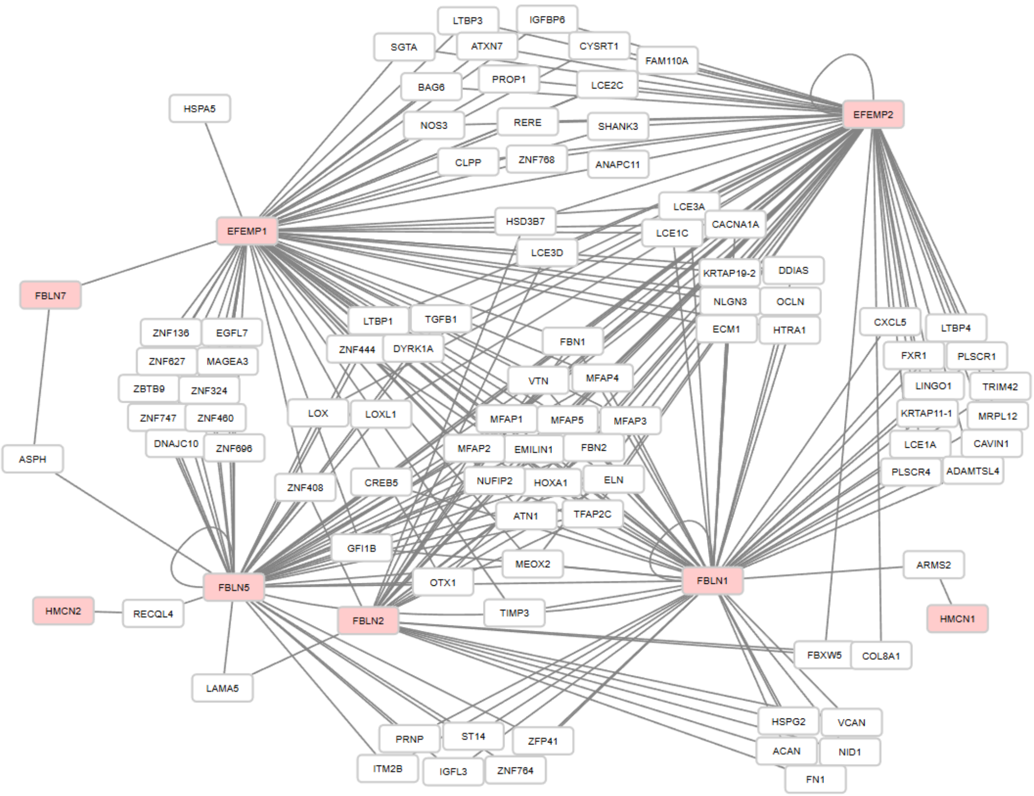

Fig. S1. Network of protein-protein interactions (PPIs). Visualization was performed with the Cytoscape tool (Shannon et al., 2003) and shows the 97 PPIs shared by at least two human fibulins (red nodes). *Legend :* HMCN1 : hemicentin-1, HMCN2 : hemicentin-2, FBLN2 : fibulin-2, FBLN1 : fibulin-1, FBLN7 : fibulin-7, FBLN5 : fibulin-5, FBLN4 : fibulin-4 (EFEMP2 gene) and FBLN3 : fibulin-3 (EFEMP1 gene). Hemicentins 1 and 2 are also known as fibulin 6 and 8 respectively.

Run

The following bash script can be used to launch the analysis, using as inputs the three files presented above ("fibulin_59.fasta", "ppi_shared.csv" and "fibulin_tree_root.tree"):

```
#!/bin/bash

# File paths
image_path="fuse-phylotree.sif"
file_fasta="fibulin_59.fasta"
file_annotation="ppi_shared.csv"
gene_tree="fibulin_tree_root.tree"
output_dir="dir_analysis_fuse_phylotree"

# Command to launch tool
cmd="python3 /fuse-phylotree/fuse-phylotree.py
--output_directory ${output_dir}
$file_fasta $file_annotation $gene_tree"

# Start analysis
singularity exec ${image_path} ${cmd}
```

where `dir_analysis_fuse_phylotree` specifies the directory where the intermediate files of the workflow are saved for advanced users.

Output

The main outputs are located in the working directory:

- The file named `0_gene.tree` (Newick format - Supplementary Files) is the gene tree with the canonical renaming of genes used by FUSE-PhyloTree.
- The file named `1_modules_and_functions_evolution.csv` (Supplementary Files) details the modules and functions assigned by FUSE-PhyloTree to each (ancestral or observed) gene, along with a view of the modules and functions lost or gained by the gene with respect to its closest ancestor in the tree. For modules, the frequency of their presence, gain, and loss prediction at the gene across 10 runs is also provided.
- The file named `2_module_descriptions.csv` (Supplementary Files) provides for each module the location (protein ID, start and end positions in the protein sequence) of its segments.
- Finally, a directory named `3_visuReconc` (Supplementary Files) gathers all the files required for viewing the genes, modules and functions evolution using iTOL.
- An additional file for expert users named `X_module_function_assoc_stats.csv` (Supplementary Files) provides co-presence statistics for all module-function pairs.

Table S2. Table of 14 nodes with module-PPI co-emergence. For each node, its (i) name (Gene), (ii) human descendants, (iii) gained PPI and (iv) gained modules with their gain frequency if below 100%, are given. Ancestral nodes highlighted in bold have at least two human paralogs as descendants.

Gene	Human descendant	PPIs gained	Modules gained
G18	hemicentin-1	ARMS2	B268 B269 B282 B290 B327 B338 B365 B372 B374 B413 B418 B419 B435 B439 B441 B444 B451 B453 B457 B463 B465 B482 B484 B492 B502 B68 (10%) B447 (90%)
G35	fibulin-1	ARMS2 CREB5 FBLN1 FBLN5 IGFL3 ITM2B PRNP ST14 ZFP41 ZNF444 ZNF764	B615 B653 B657 B681
G37	fibulin-1	CACNA1A GFI1B LCE1C LCE3A MEOX2	B677 B684 B686 B610 (10%)
G46	fibulin-2	HSD3B7 LAMA5 LCE3D	B615 B687 B701 B703 B705 B714 B880
G47	fibulin-2	OTX1	B697 B713 B715
**G50**	**fibulin-1,-2**	**ACAN FN1 HSPG2 NID1 VCAN**	**B663 B674 B679 B680 B728 B730 B736 B737 B739 B741 B49 (10%) B68 (10%) B565 (20%) B610 (10%) B727 (10%)**
G68	fibulin-7	ASPH	B745 B746
G82	fibulin-5	ASPH	B878
G93	fibulin-4	ADAMTSL4 CAVIN1 COL8A1 CXCL5 EFEMP2 FBXW5 FXR1 KRTAP111 LCE1A LINGO1 LOX LOXL1 LTBP4 MRPL12 PLSCR1 PLSCR4 TRIM42	B64 (10%)
G94	fibulin-4	CREB5 FBLN5	B615 B648 B651
G109	fibulin-3	CACNA1A HSD3B7 LCE1C LCE3A LCE3D	B244
**G110**	**fibulin-3,-4**	**ANAPC11 ATXN7 BAG6 CLPP CYSRT1 FAM110A IGFBP6 LCE2C LTBP3 NOS3 PROP1 RERE SGTA SHANK3 ZNF768**	**B593 B617 B629 B249 (10%)**
**G111**	**fibulin-3,-4,-5**	**DYRK1A GFI1B LTBP1 MEOX2 OTX1 TGFB1**	**B595 B612 B622 B624 B626 B628 B644 B68 (10%) B565 (20%) B610 (20%) B625 (60%)**
**G115**	**fibulin-1,-2,-3,-4,-5,-7**	**ATN1 ELN EMILIN1 FBN1 FBN2 HOXA1 MFAP1 MFAP2 MFAP3 MFAP4 MFAP5 NUFIP2 TFAP2C VTN**	**B56 B643 B769 B883 B27 (10%) B610 (80%)**

For our use case involving 59 fibulin sequences, FUSE-PhyloTree completed the analysis in under 115 minutes, using Docker or Singularity containers on a laptop (64 GB memory, Intel® Core™ Ultra 7 165U CPU with 14 threads). As a result, the file `2_module_descriptions.csv` lists 508 modules. Their inferred co-evolution with functions can be examined by looking at the file `1_modules_and_functions_evolution.csv`. It enables, for instance, to distinguish among the 58 ancestral genes of the phylogeny, 51 ancestral genes with a gain of modules and, among them, 14 ancestral genes with the co-emergence of at least one module and one PPI (see Table S2). Among these 14 ancestral genes, 4 have at least two

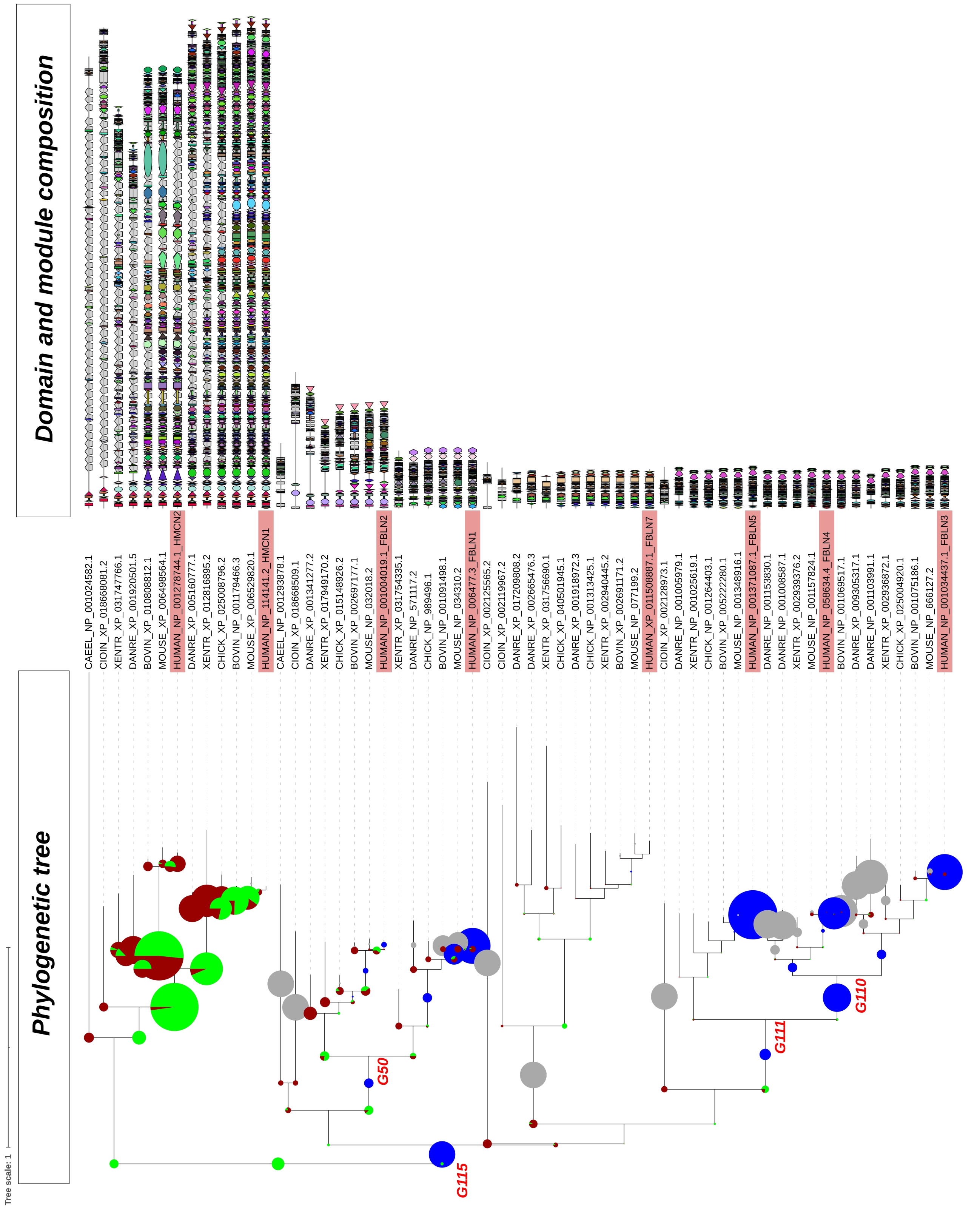

Fig. S2. Representation on a phylogenetic tree of the modules and PPIs emergence. The phylogenetic tree is shown with the number of gains and losses of PPIs (blue and grey circles, respectively) and of modules (green and red respectively in the pie chart) at the ancestor nodes. Identifiers of leaf sequences are preceded by species abbreviations: CAEEL, *Caenorhabditis elegans*; CIOIN, *Ciona intestinalis*; DANRE, *Danio rerio*; XENTR, *Xenopus tropicalis*; CHICK, *Gallus gallus*; BOVIN, *Bos taurus*; MOUSE, *Mus musculus*; HUMAN, *Homo sapiens*. Leaf labels of human fibulins are highlighted in red and followed by paralog abbreviations: HMCN1, hemicentin-1; HMCN2, hemicentin-2; FBLN2, fibulin-2; FBLN1, fibulin-1; FBLN7, fibulin-7; FBLN5, fibulin-5; FBLN4, fibulin-4 (*EFEMP2*); and FBLN3, fibulin-3 (*EFEMP1*). Hemicentins 1 and 2 are also known as fibulins 6 and 8, respectively. In the domain and module composition section, each module is represented by a unique shape and color combination, while grey background shapes indicate the positions of Pfam domains. The presence of large red and green pie charts in the hemicentins' subtree is due to their greater length compared to other fibulins, resulting in more modules. In contrast, the presence of blue and grey pie charts in the subtrees of fibulins 3, 4, 5, and of fibulin 1 reflects the high number of known PPIs for these human proteins.

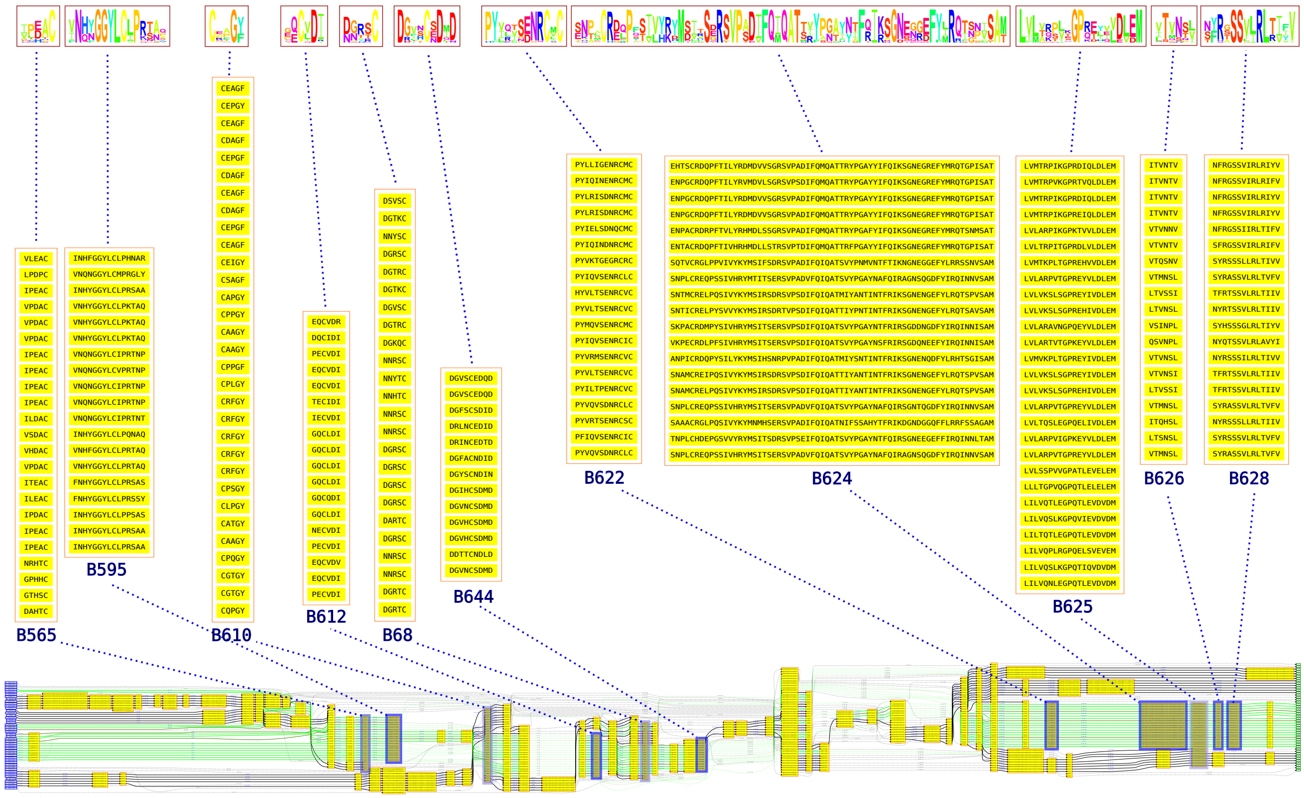

Fig. S3. Modules gained at G111 on fibulins. Hemicentin sequences being too long to display, only fibulin sequences from G115 subtree are considered here. The partial local multiple alignment (PLMA) of the sequences used to identify the conserved modules is shown at the bottom of the figure. Set of conserved segments are highlighted in yellow and surrounded by red boxes. Many conserved segment sets involve only a few sequences, but only those involving at least 5 segments are displayed here. Lines indicate the path of each sequence between its conserved segments, with green colors used for descendant sequences from G111 (dark green specifically representing human sequences). Modules predicted to be gained at ancestor gene G111 (B565, B595, B610, B612, B622, B624, B625, B626, B628, B644 and B68) are highlighted in blue in the PLMA with a zoom on their segment content above and, on the top of the figure, a sequence logo (Crooks et al., 2004) showing their positional residue conservation.

human descendants: G50, G110, G111 and G115. Note that, in addition to their importance in identifying relevant conserved modules, orthologs enable FUSE-PhyloTree to predict the co-emergence of modules and functions of ancestral genes with a single human descendant, for instance the ancestral gene G94 of fibulin 4, which gains two PPIs, CREB5 and FBLN5 and 3 conserved modules B615, B648 and B651. Fig. S2 shows an iTOL visualization of the results, obtained from the files available in `3_visuReconc` directory. The interactive version of the tree is available at `https://itol.embl.de/tree/13125423118169031752734489`

Interpretation

To illustrate the exploration of the results, we propose to zoom in on the gene G111 from Table S2, the common ancestor of three human fibulins (FBLN3, FBLN4 and FBLN5) called the elastic fibulins.

G111 is predicted to gain 6 interactions (with DYRK1A, GFI1B, LTBP1, MEOX2, OTX1 and TGFB1), 3 of which (DYRK1A, LTBP1 and TGFB1) are specific to this subtree. Interestingly, LTBP1 and TGFB1 are biologically closely related, as LTPB1 is known to bind the latent form of TGFB1. It can be noted that DYRK1A has also connections to TGFB1, as it has been shown to interact with TGFB1 signaling pathway (Cao et al., 2025).

Alongside its gained interactions, G111 is predicted to gain 11 modules (B595, B612, B622, B624, B626, B628, B644 with 100% frequency, B625 with 60% frequency, B565 and B610 with 20% frequency and B68 with 10% frequency) shown in Fig. S3. Let's remark that modules with low gain frequency are shared with sequences other than the G111 descendants, but are still predominantly found in those descendants. Looking at the human G111 descendants, 9 of the gained modules are present in all the three sequences. The two other modules B68 and B644 are absent in FBLN3, but are present in orthologs of FBLN3 (B68 in *Xenopus tropicalis*, *Mus musculus*, *Gallus gallus*, *Danio rerio* and *Bos taurus* and B624 in *Gallus gallus* and *Mus musculus*), supporting FUSE-PhyloTree prediction that they were gained in G111 and subsequently lost in specific lineages.

The locations of the conserved segments of these modules in the sequences of G111's human descendants are shown in Fig. S4 (see also Fig. S5).

The modules gained at G111 predominantly span the characteristic C-terminal domain of fibulins, detected by "Fibulin C-terminal Ig-like" PFAM model (PF22914, Mistry et al., 2020). Modules B624, B625 B626 and B628 cover this PFAM domain and enable to refine its functional role

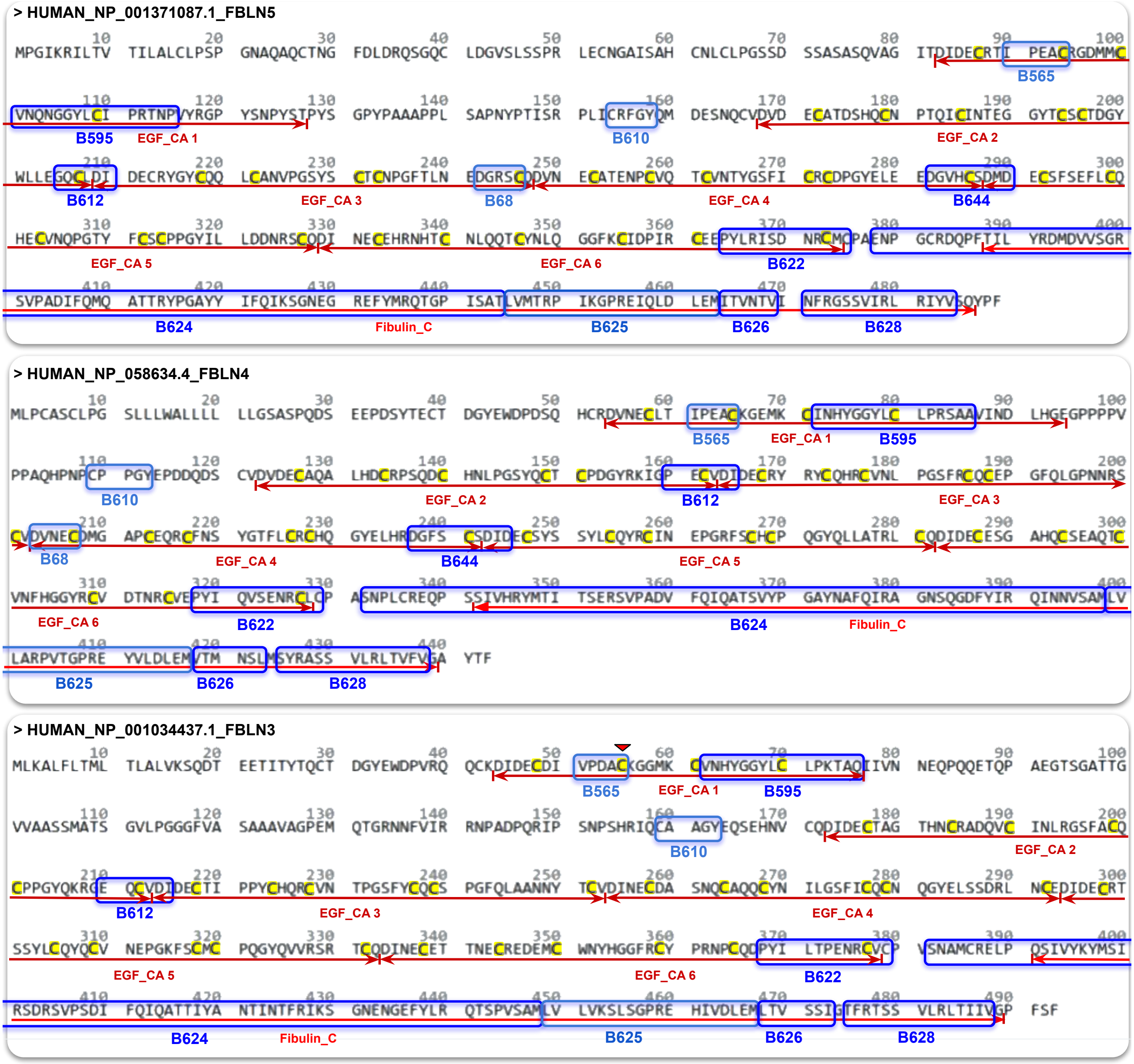

Fig. S4. Position of modules gained by the G111 ancestral gene in its human descendants. Segments of modules gained at G111 (B565, B595, B610, B612, B622, B624, B625, B626, B628, B644 and B68) are shown here circled by blue boxes, with their name underneath. Note that modules B68 and B644 are not conserved in FBLN3. The six occurrences of SMART SM0017 domain (EGF_CA, numbered from 1 to 6 in each sequence) and the occurrence of PFAM PF22914 domain (Fibulin_C) in the sequences are underlined by red arrows, with their name underneath. Conserved cysteine residues in EGF_CA domains are highlighted in yellow and FBLN3's cysteine residue at position 55 is marked by red triangle.

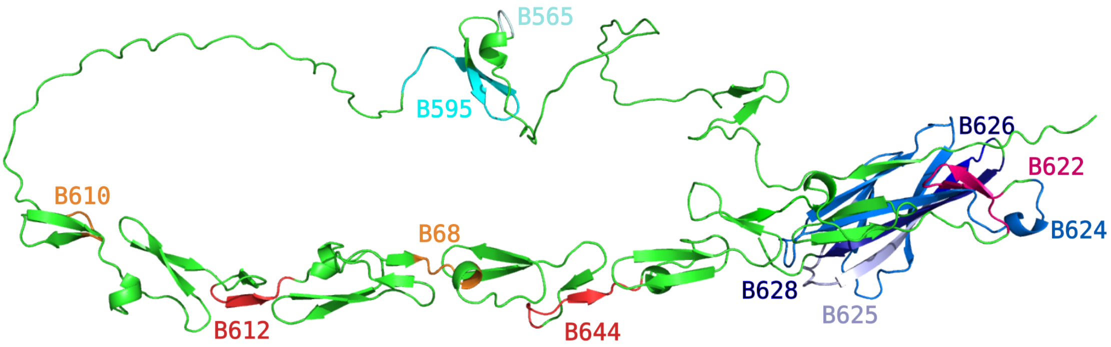

Fig. S5. Location of G111 gained modules on predicted 3D structure of HUMAN_NP_001371087.1_FBLN5 by AlphaFold (AF-G3XA98-F1-v4, Varadi et al., 2023).

by revealing a specific conservation of the region in FBLN 3, 4 and 5 subtree that is probably linked to their specific interactions. This prediction was confirmed for LTBP1 by experimental studies showing the interaction of the C-terminal domain of FBLN4 and FBLN5 with LTBP proteins (Noda et al., 2013; Hirai et al., 2007). Additionally, module B624 extends beyond the identified PFAM domain, suggesting an expanded functional region of interest.

Module B610 is an interesting exception, as it does not map to any known domain or motif. The remaining gained modules (B565, B595, B612, B68, B644 and B622) map to calcium-binding EGF-like (EGF_CA) domains of fibulins detected by SMART (SM0017, Letunic et al., 2020). Modules B565 and B595 are included in EFG_CA 1, an atypical EFG_CA domain (with two of the six conserved cysteine residues of the domain missing) that is nonetheless functionally important, as shown by the study of the mutation of FBLN3 cysteine residue at position 55 (Woodard et al., 2022) that is in B565. Modules B612, B68, B644, and B622 are found at the end of EGF_CA domains 2, 3, 4, and 6, respectively. Interestingly, each is positioned around the last cysteine residue of the domain, in its exposed loop and second strand of C-terminal β-sheet region (see Fig S5). It can also be noted that module B622 is adjacent to the extension of the PFAM domain by B624 in the 3D structure, and that module B610 occupies a similar 3D location to module B68. Combined with their residue composition shown in Fig. S3, in particular the presence of negatively charged conserved residues, this reinforces their potential role in interaction specificity and makes them interesting candidates for therapeutic targeting, which merits further investigation.

To conclude, analysis of the fibulin family using FUSE-PhyloTree revealed the evolutionary history of 508 conserved modules and 97 PPIs. It identified 14 ancestral genes for which conserved modules and PPI co-emerged. The interpretation of these predictions was illustrated for the ancestral gene G111, demonstrating that FUSE-PhyloTree could identify modules involved in experimentally validated interactions and also reveal novel potential functional modules. Beyond these direct predictions, FUSE-PhyloTree can also be used to interactively explore the evolution of functions and sequence conservation along the phylogenetic gene tree, and gain deeper insights into the family. For example, it would be interesting to explore with iTOL the direct ancestors and descendants of the gene G111 to refine predictions, or entire paths to G111, or from G111, to obtain information on the evolution of modules and their specificity.

All the files resulting from the analysis are available on GitHub at (`https://github.com/OcMalde/fuse-phylotree/tree/main/data/analyse_fibulin`).

References

B. Aranda et al. PSICQUIC and PSISCORE: Accessing and scoring molecular interactions. *Nature Methods*, 8(7):528–529, July 2011. ISSN 1548-7105. doi: 10.1038/nmeth.1637.

Y. Cao et al. DYRK1A-TGF-β signaling axis determines sensitivity to OXPHOS inhibition in hepatocellular carcinoma. *Developmental Cell*, pages S1534-5807(24)00775–5, Jan. 2025. ISSN 1878-1551. doi: 10.1016/j.devcel.2024.12.035.

M. A. Cooley and W. S. Argraves. The Fibulins. In R. P. Mecham, editor, *The Extracellular Matrix: An Overview*, pages 337–367. Springer, Berlin, Heidelberg, 2011. ISBN 978-3-642-16555-9. doi: 10.1007/978-3-642-16555-9_10.

G. E. Crooks, G. Hon, J.-M. Chandonia, and S. E. Brenner. Weblogo: A sequence logo generator. *Genome Research*, 14 (6):1188–1190, 2004. doi: 10.1101/gr.849004. URL `http://genome.cshlp.org/content/14/6/1188.abstract`.

W. M. Gallagher, C. A. Currid, and L. C. Whelan. Fibulins and cancer: Friend or foe? *Trends in Molecular Medicine*, 11(7):336–340, July 2005. ISSN 1471-4914. doi: 10.1016/j.molmed.2005.06.001.

M. Hirai et al. Latent TGF-beta-binding protein 2 binds to DANCE/fibulin-5 and regulates elastic fiber assembly. *The EMBO journal*, 26(14):3283–3295, July 2007. ISSN 0261-4189. doi: 10.1038/sj.emboj.7601768.

I. Letunic and P. Bork. Interactive Tree of Life (iTOL) v6: Recent updates to the phylogenetic tree display and annotation tool. *Nucleic Acids Research*, 52(W1):W78–W82, July 2024. ISSN 0305-1048. doi: 10.1093/nar/gkae268.

I. Letunic, S. Khedkar, and P. Bork. Smart: recent updates, new developments and status in 2020. *Nucleic Acids Research*, 49(D1):D458–D460, 10 2020. ISSN 0305-1048. doi: 10.1093/nar/gkaa937. URL `https://doi.org/10.1093/nar/gkaa937`.

D. Mahajan et al. Role of Fibulins in Embryonic Stage Development and Their Involvement in Various Diseases. *Biomolecules*, 11(5):685, May 2021. ISSN 2218-273X. doi: 10.3390/biom11050685.

J. Mistry et al. Pfam: The protein families database in 2021. *Nucleic Acids Research*, 49(D1):D412–D419, 10 2020. ISSN 0305-1048. doi: 10.1093/nar/gkaa913. URL `https://doi.org/10.1093/nar/gkaa913`.

K. Noda et al. Latent TGF-β binding protein 4 promotes elastic fiber assembly by interacting with fibulin-5. *Proceedings of the National Academy of Sciences of the United States of America*, 110(8):2852–2857, Feb. 2013. ISSN 1091-6490. doi: 10.1073/pnas.1215779110.

I. B. Robertson et al. Latent TGF-β-binding proteins. *Matrix Biology: Journal of the International Society for Matrix Biology*, 47:44–53, Sept. 2015. ISSN 1569-1802. doi: 10.1016/j.matbio.2015.05.005.

P. Shannon et al. Cytoscape: A Software Environment for Integrated Models of Biomolecular Interaction Networks. *Genome Research*, 13(11):2498–2504, Nov. 2003. ISSN 1088-9051, 1549-5469. doi: 10.1101/gr.1239303.

M. Varadi et al. Alphafold protein structure database in 2024: providing structure coverage for over 214 million protein sequences. *Nucleic Acids Research*, 52(D1):D368–D375, 11 2023. ISSN 0305-1048. doi: 10.1093/nar/gkad1011. URL `https://doi.org/10.1093/nar/gkad1011`.

D. R. Woodard et al. A loss-of-function cysteine mutant in fibulin-3 (efemp1) forms aberrant extracellular disulfide-linked homodimers and alters extracellular matrix composition. *Human Mutation*, 43(12):1945–1955, 2022. doi: https://doi.org/10.1002/humu.24452. URL `https://onlinelibrary.wiley.com/doi/abs/10.1002/humu.24452`.
